# Initial empirical antibiotic therapy in kidney transplant recipients with pyelonephritis: A global survey of current practice and opinions across 19 countries on six continents

**DOI:** 10.1111/tid.14362

**Published:** 2024-08-26

**Authors:** Julien Coussement, Shyam B. Bansal, Anne Scemla, My H. S. Svensson, Laura A. Barcan, Olivia C. Smibert, Wanessa T. Clemente, Francisco Lopez‐Medrano, Tomer Hoffman, Umberto Maggiore, Concetta Catalano, Luuk Hilbrands, Oriol Manuel, Tinus DU TOIT, Terence Kee Yi Shern, Nizamuddin Chowdhury, Ondrej Viklicky, Rainer Oberbauer, Samuel Markowicz, Hannah Kaminski, Matthieu Lafaurie, Ligia C. Pierrotti, Tiago L. Cerqueira, Dafna Yahav, Nassim Kamar, Camille N. Kotton

**Affiliations:** ^1^ Department of Infectious Diseases Guadeloupe University Hospital Les Abymes France; ^2^ Sir Peter MacCallum Department of Oncology University of Melbourne Melbourne Australia; ^3^ Department of Nephrology Medanta‐Medicity Gurgaon India; ^4^ Department of Kidney Transplantation Hôpital Necker‐Enfants Malades, Assistance Publique – Hôpitaux de Paris Paris France; ^5^ Department of Nephrology Aalborg University Hospital Aalborg Denmark; ^6^ Internal Medicine Department Infectious Diseases Section Hospital Italiano de Buenos Aires Buenos Aires Argentina; ^7^ Department of Infectious Diseases Austin Health Heidelberg Australia; ^8^ Department of Laboratory Medicine Transplant Program Hospital das Clínicas‐Universidade Federal de Minas Gerais (UFMG) School of Medicine (UFMG) Belo Horizonte Brazil; ^9^ Department of Medicine, Unit of Infectious Diseases, Hospital Universitario “12 de Octubre”, Instituto de Investigación Sanitaria Hospital “12 de Octubre” (imas12), Centro de Investigación Biomédica en Red de Enfermedades Infecciosas (CIBERINFEC), Instituto de Salud Carlos III (ISCIII), School of Medicine Universidad Complutense Madrid Spain; ^10^ Infectious Diseases Unit, Sheba Medical Center Tel Hashomer Israel; ^11^ Department of Medicine and Surgery Kidney‐Pancreas Transplant Unit University of Parma Parma Italy; ^12^ Department of Nephrology Dialysis and Kidney Transplantation, CUB‐Hôpital Erasme Brussels Belgium; ^13^ Department of Nephrology Radboud University Medical Center Nijmegen The Netherlands; ^14^ Transplantation Centre and Service of Infectious Diseases University Hospital of Lausanne Lausanne Switzerland; ^15^ Transplant Unit Groote Schuur Hospital and University of Cape Town Cape Town South Africa; ^16^ Department of Renal Medicine Singapore General Hospital Singapore Singapore; ^17^ Department of Nephrology BRB Hospitals Ltd Dhaka Bangladesh; ^18^ Department of Nephrology Institute for Clinical and Experimental Medicine Prague Czech Republic; ^19^ Division of Nephrology Department of Internal Medicine III Medical University of Vienna Vienna Austria; ^20^ Department of Nephrology Transplantation, Dialysis and Apheresis, Pellegrin University Hospital Bordeaux France; ^21^ Infectious Diseases Unit St‐Louis Hospital Assistance Publique – Hôpitaux de Paris Paris France; ^22^ Infectious Diseases Division Hospital das Clínicas University of São Paulo Medical School Sao Paulo Brazil; ^23^ Department of Kidney Transplant Hospital Evangelico de Minas Gerais Belo Horizonte Brazil; ^24^ Department of Nephrology and Organ Transplantation Toulouse Rangueil University Hospital, Toulouse Institute for Infectious and Inflammatory Diseases (Infinity), University Paul Sabatier Toulouse France; ^25^ Transplant Infectious Disease and Compromised Host Program Division of Infectious Diseases Massachusetts General Hospital Boston Massachusetts USA; ^26^ Department of Medicine Harvard Medical School Boston Massachusetts USA

**Keywords:** antimicrobial stewardship, kidney transplantation, questionnaire, urinary tract infection

## Abstract

**Background:**

Despite the burden of pyelonephritis after kidney transplantation, there is no consensus on initial empirical antibiotic management.

**Methods:**

We surveyed clinicians throughout the world on their practice and opinions about the initial empirical therapy of post‐transplant pyelonephritis, using clinical vignettes. A panel of experts from 19 countries on six continents designed this survey, and invited 2145 clinicians to participate.

**Results:**

A total of 721 clinicians completed the survey (response rate: 34%). In the hypothetical case of a kidney transplant recipient admitted with pyelonephritis but not requiring intensive care, most respondents reported initiating either a 3rd‐generation cephalosporin (37%) or piperacillin‐tazobactam (21%) monotherapy. Several patient‐level factors dictated the selection of broader‐spectrum antibiotics, including having a recent urine culture showing growth of a resistant organism (85% for extended‐spectrum ß‐lactamase‐producing organisms, 90% for carbapenemase‐producing organisms, and 94% for *Pseudomonas aeruginosa*). Respondents attributed high importance to the appropriateness of empirical therapy, which 87% judged important to prevent mortality. Significant practice and opinion variations were observed between and within countries.

**Conclusion:**

High‐quality studies are needed to guide the empirical management of post‐transplant pyelonephritis. In particular, whether prior urine culture results should systematically be reviewed and considered remains to be determined. Studies are also needed to clarify the relationship between the appropriateness of initial empirical therapy and outcomes of post‐transplant pyelonephritis.

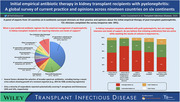

## Graphical abstract

Pyelonephritis is common after kidney transplantation. Experts from 19 countries on six continents surveyed clinicians on their practice and opinions about the initial empirical therapy of post‐transplant pyelonephritis (i.e., prior to the receipt of culture results). A total number of 721 clinicians completed the survey (response rate: 34%).

**Abbreviation**
ESBL: extended‐spectrum ß‐lactamase

## 1 INTRODUCTION

Despite the high frequency and negative consequences of acute pyelonephritis after kidney transplantation,^1^, ^2^, ^3^ many management questions remain understudied and therefore unanswered.^4^ One of these questions is what empirical antibiotic therapy should be initiated in kidney transplant recipients presenting with symptoms and signs of pyelonephritis.

While experts have suggested that empirical therapy for pyelonephritis should provide ≥ 90% likelihood of in vitro activity against the causal pathogen,^5^, ^6^ the relevance and applicability of this rule are unknown in kidney transplant recipients. To reach this target in the era of emerging antimicrobial resistance, many would need to systematically initiate very broad‐spectrum antibiotics (e.g., carbapenems) or combination therapy in kidney transplant recipients presenting with pyelonephritis.^7^ However, information on the effects of inappropriate empirical therapy in kidney transplant recipients with pyelonephritis is missing, especially for patients who do not require intensive care unit levels of support. While there are data demonstrating that solid organ transplant recipients with an infection requiring hospital admission have higher mortality when appropriate empirical therapy is delayed,^8^ this may not be true in kidney transplant recipients with pyelonephritis, in whom mortality rates are relatively low.^9^, ^10^, ^11^ Also, microbiological documentation is available in virtually all episodes of pyelonephritis, enabling antibiotic therapy to be adjusted after 1–2 day(s) if initially inappropriate.

Despite the burden of pyelonephritis after kidney transplantation, very few data are available to guide clinicians managing kidney transplant recipients with pyelonephritis.^2^ Local guidelines may not be available or cover this question adequately and, in many countries, there are no guidelines specifically addressing the empirical management of post‐transplant pyelonephritis.

We designed this international survey to understand practices and opinions regarding the initial empirical management of post‐transplant pyelonephritis.

## 2 MATERIAL AND METHODS

### 2.1 Survey content

A panel of 26 experts from 19 countries on six continents (Europe, North America, South America, Oceania, Asia, and Africa) designed this questionnaire‐based, online survey looking at the empirical antibiotic therapy of acute pyelonephritis in adult kidney transplant recipients. The term ‘empirical’ therapy refers to the initiation of antibiotic therapy in a patient presenting with probable pyelonephritis (i.e., prior to the receipt of culture and susceptibility testing results). In contrast, the term ‘definitive’ therapy refers to the therapy given after receipt of these microbiological results. The survey content was based on literature review^3^ and adapted by consensus to require approximately 5–10 min for completion. A paper version of the questionnaire can be found in the Appendix pp 6–9. Briefly, the questionnaire included five questions concerning participants’ characteristics and six questions on their opinions and management of post‐transplant pyelonephritis. Hypothetical clinical vignettes were used. This web‐based survey was created using SurveyMonkey and pretested by survey coordinators for accuracy and functionality. Ethics committee approval was deemed unnecessary for this survey, as it only collected the personal opinions of clinicians and did not contact patients or require any specific patient data.

### 2.2 Survey participants

Our target population was clinicians involved in the care of adult kidney transplant recipients, including not only nephrologists but also infectious disease physicians and transplant surgeons. To improve survey representativeness, there was at least one national coordinator for each of the 19 participating countries; these renowned experts were asked to invite, in their country, a representative sample of clinicians involved in the care of adult kidney transplant recipients. Globally, 2145 clinicians were invited to participate, through an e‐mail sent in English. No money was offered for survey participation. The number of responses per hospital were not limited. One reminder e‐mail was sent to increase the survey response rate. The survey was open online between November 20 and December 24, 2023.

### 2.3 Data collection and statistical analysis

The response rate was defined as the ratio of the number of respondents to the total number of invitees. Responses in which only the first section of the survey (i.e., the section on respondents’ characteristics) was completed were discarded; all other responses were included. Results are expressed as numbers and percentages for categorical variables, and median and interquartile ranges for continuous variables.

## 3 RESULTS

### 3.1 Survey response rate and respondents’ characteristics

A total of 721 clinicians from 19 countries on six continents completed the survey, representing a response rate of 34% (721/2145). Additionally, nine responses in which only the first section of the survey (i.e., the section on respondents’ characteristics) was completed were discarded. Countries that contributed the most were India (*n* = 140), France (*n* = 90), the United States (*n* = 83), Denmark (*n* = 45), and Argentina (*n* = 42; details in Table S1). Characteristics of survey respondents are presented in Table S2. In summary, most respondents were nephrologists (68%, 491/721) followed by infectious diseases physicians (29%, 208/721). Overall, 83% were specialists for at least five years (600/721) and 76% worked in an academic hospital (551/721).

### 3.2 Preferred antibiotic regimen for the initial empirical management of post‐transplant pyelonephritis

**FIGURE 1:**
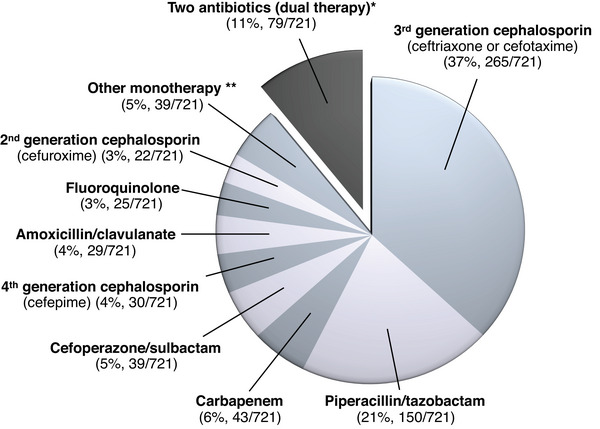
A kidney transplant recipient is admitted to your hospital with fever, dysuria, and graft pain, and is diagnosed with probable graft pyelonephritis. Two sets of blood cultures and a urine sample are collected, and you then decide to initiate empirical antibiotic therapy while awaiting the results of the cultures. The patient does not require intensive care levels of support, and there are no recent culture results to guide antibiotic choice. What is your preferred empirical antibiotic regimen?

In the hypothetical case of a kidney transplant recipient admitted with probable pyelonephritis but not requiring intensive care levels of support, 89% of respondents (642/721) selected a single‐drug regimen (Figure 1). Preferred single‐drug regimens were a 3^rd^ generation cephalosporin such as ceftriaxone or cefotaxime (37%, 265/721), piperacillin‐tazobactam (21%, 150/271), or a carbapenem (6%, 43/721).

Conversely, 11% of survey respondents (79/721) declared systematically initiating a combination of two antibiotics in this clinical situation. Preferred combinations were those associating a ß‐lactam with a fluoroquinolone (3%, 24/721), a ß‐lactam with an aminoglycoside (3%, 20/721), or a ß‐lactam with a glycopeptide (2%, 11/721).

### 3.3 Patient‐level factors influencing prescribing behaviors

As shown in Table 1, several clinical scenarios significantly increased respondents’ propensity to initiate a broader‐spectrum empirical therapy, e.g., presenting with sepsis or septic shock (85%, 592/698), having a recent urine culture with growth of a resistant organism (85% for extended‐spectrum ß‐lactamase [ESBL]‐producing organisms, 596/698), having recently received an antibiotic treatment (57%, 397/698) or having a healthcare‐associated episode of pyelonephritis (defined as urinary catheter, hospital stay and/or hemodialysis in last 3–6 months; 57%, 394/698).

**TABLE 1:** Reasons to select an initial empirical regimen that has a broader spectrum than the one selected in standard episodes of post‐transplant pyelonephritis.

CLINICAL VIGNETTE: A kidney transplant recipient is admitted to your hospital with fever, dysuria, and graft pain, and is diagnosed with probable graft pyelonephritis. Two sets of blood cultures and a urine sample are collected, and you then decide to initiate empirical antibiotic therapy while awaiting the results of the cultures. The patient does not require intensive care levels of support, and there are no recent culture results to guide antibiotic choice.	
**Would any of the following scenarios make you select a regimen that has a broader spectrum than the standard regimen you selected in the previous question (see** **Figure** **1** **)? (n = 698)**	
Septic shock or sepsis Imaging done at admission showed urinary tract obstruction Imaging done at admission showed an abscess of the kidney graft Recent transplant (last 3–6 months) Healthcare‐associated infection^*^ Recent antibiotic treatment (last 3–6 months) Current use of trimethoprim/sulfamethoxazole prophylaxis Current use of another antibiotic to prevent urinary tract infections^**^ Recent urine culture with growth of ESBL‐producing organism (last 3–6 months) Recent urine culture with growth of carbapenemase‐producing organism (last 3–6 months)	85% (*n* = 592) 29% (*n* = 199) 57% (*n* = 395) 30% (*n* = 207) 57% (*n* = 394) 57% (*n* = 397) 14% (*n* = 97) 36% (*n* = 252) 85% (*n* = 596) 90% (*n* = 628)

*n*: number of answers received.

* Defined as urinary catheter, hospital stay, and/or hemodialysis in last 3–6 months.

** e.g., nitrofurantoin, fosfomycin, or cephalexin. ESBL: extended‐spectrum ß‐lactamase.

### 3.4 Opinions about the need to empirically cover *Pseudomonas aeruginosa* and *Enterococcus* spp. in kidney transplant recipients with pyelonephritis

Overall, only a minority of respondents reported systematically covering *Pseudomonas aeruginosa* and *Enterococcus* spp. in all kidney transplant recipients presenting with pyelonephritis (26% [175/681] and 14% [97/672], respectively). However, several patient‐level factors increased the respondents’ propensity to empirically cover *P. aeruginosa* and *Enterococcus* spp. (Table 2). The strongest of these patient‐level factors was having a recent urine culture showing growth of *P. aeruginosa* or *Enterococcus* spp., which prompted the vast majority of clinicians to cover these organisms at the time of pyelonephritis (94% [642/681] for *P. aeruginosa* and 85% [573/672] for *Enterococcus* spp.).

**TABLE 2:** Answers obtained regarding the importance of empirically covering *Pseudomonas aeruginosa* and *Enterococcus* spp. in kidney transplant recipients who present with pyelonephritis but do not require intensive care unit admission.

Should empirical therapy cover *Pseudomonas aeruginosa* in kidney transplant recipients who present with symptoms of pyelonephritis (e.g., fever, dysuria, and graft pain) but do not require intensive care unit admission? (*n* = 681)	
Yes (systematically for all hospitalized patients) No (never) In the following situation(s):	26% (*n* = 175) 6% (*n* = 38)
Recent urine culture with significant growth of *P. aeruginosa* (last 3–6 months) Imaging at admission showing urinary tract obstruction or graft abscess Kidney transplantation in the last 3–6 months Healthcare‐associated infection^*^ Receipt of antibiotic therapy in the last 3–6 months Current receipt of trimethoprim/sulfamethoxazole prophylaxis Current receipt of another antibiotic to prevent urinary tract infections^**^	94% (*n* = 642) 44% (*n* = 302) 37% (*n* = 251) 57% (*n* = 390) 43% (*n* = 294) 29% (*n* = 196) 36% (*n* = 248)
**Should empirical therapy cover *Enterococcus* spp. in kidney transplant recipients who present with symptoms of pyelonephritis (e.g., fever, dysuria, and graft pain) but do not require intensive care unit admission? (*n* = 672)**	
Yes (systematically for all hospitalized patients) No (never) In the following situation(s):	14% (*n* = 97) 16% (*n* = 109)
Recent urine culture with significant growth of Enterococcus spp. (last 3–6 months) Imaging at admission showing urinary tract obstruction or graft abscess Kidney transplantation in the last 3–6 months Healthcare‐associated infection ^*^ Receipt of antibiotic therapy in the last 3–6 months Current receipt of trimethoprim/sulfamethoxazole prophylaxis Current receipt of another antibiotic to prevent urinary tract infections ^**^	85% (*n* = 573) 29% (*n* = 193) 22% (*n* = 145) 31% (*n* = 211) 24% (*n* = 160) 18% (*n* = 118) 21% (*n* = 143)

n: number of answers received.

* Defined as urinary catheter, hospital stay, or hemodialysis in last 3–6 months.

** e.g., nitrofurantoin, fosfomycin, or cephalexin.

### 3.5 Perceived importance of the appropriateness of initial empirical therapy in post‐transplant pyelonephritis

**FIGURE 2:**
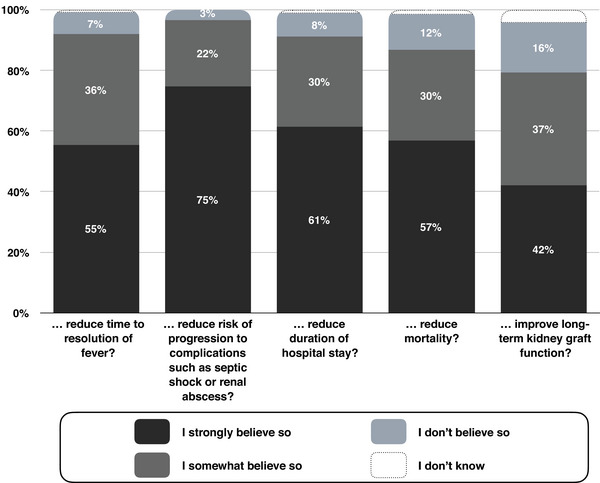
In kidney transplant recipients admitted to your hospital with probable graft pyelonephritis but not requiring intensive care levels of support, do you believe that initiating antibiotic(s) that are active while awaiting the results of cultures is important to....

Survey participants were asked about the importance of the appropriateness of initial empirical antibiotic therapy in non‐severe patients (i.e., in kidney transplant recipients who are admitted with probable pyelonephritis, but do not require intensive care unit levels of support). As shown in Figure 2, the vast majority of survey respondents declared that the appropriateness of initial empirical antibiotic therapy was important to prevent major complications, including the risk of progression to septic shock or renal abscess (96%, 632/655), and the risk of death (87%, 568/655). Furthermore, respondents estimated at around 8% the risk of 30‐day mortality in patients initially receiving inappropriate empirical therapy but then receiving appropriate antibiotic therapy once urine and blood cultures results were provided (median estimated risk of death: 8%, interquartile range: 5%–20%, 636 responses obtained).

### 3.6 Variation in practice and opinions between and within countries

There was notable variability between and within countries in terms of choice of initial empirical regimen (Table S3). As an illustration, a 3rd generation cephalosporin monotherapy was selected by most participants from France, Brazil, Spain, the Netherlands, Switzerland, and Singapore, while either piperacillin‐tazobactam or cefepime (i.e., broader‐spectrum ß‐lactams that are active against *P. aeruginosa*) were selected by most participants from Denmark, Italy and the United States (Table S3). Other geographical variations included the fact that cefoperazone/sulbactam (i.e., an antibiotic with activity against *P*. aeruginosa and other multidrug‐resistant organisms) was the monotherapy of choice in India (37%, 38/103).

There was also notable variability between countries in terms of organisms systematically covered (Table S3). In particular, most respondents from India, Italy, and Bangladesh declared systematically covering *P. aeruginosa*, as compared with less than 5% of respondents from France, Belgium, the Netherlands, and Switzerland.

### 3.7 Variation in practice and opinions between nephrologists and infectious disease physicians

There was only limited variation between nephrologists (*n* = 491) and infectious diseases physicians (*n* = 208) in terms of practice and opinions (Table S4). In both groups, around 90% of respondents selected a single‐drug regimen to treat standard cases of post‐transplant pyelonephritis, generally using either a 3rd generation cephalosporin or piperacillin‐tazobactam. Both groups attributed high importance to results from prior urine cultures, as illustrated by the fact that around 95% of respondents from both groups declared systematically initiating anti‐pseudomonal antibiotics if the patient had a recent urine culture showing significant growth of *P. aeruginosa*. Last, the vast majority of respondents from both groups considered the appropriateness of initial empirical antibiotic therapy to be important to preventing death and other possible complications of pyelonephritis (Table S4).

## 4 DISCUSSION

The present survey was designed to capture current opinions and practices of clinicians involved throughout the world in the initial empirical management of post‐transplant pyelonephritis. Responses obtained from 721 clinicians from 19 countries on six continents indicated that broad‐spectrum antibiotics are commonly used in this indication, that there is wide variability between and within countries, that prescribers use many patient‐level factors of unknown relevance to guide initial antibiotic choice, and that many clinicians consider the appropriateness of empirical therapy to be critical to prevent death and other infectious complications.

We conducted this survey because pyelonephritis is common after kidney transplantation^1^ and because optimal empirical therapy selection (i.e., finding the antibiotic regimen that will be best suited to treat the infection) is difficult. On one side, initiating inappropriate therapy (i.e., administering pending culture results in a regimen that does not adequately cover the causal pathogen) may be detrimental to patient and graft outcomes.^12^ On the other side, selecting a regimen that has an excessively broad spectrum may lead to unnecessary side effects. Given that the gut microbiota is damaged very early during an antibiotic course,^13^, ^14^ overuse of broad‐spectrum empirical antibiotics −even during just a few days− may increase the risks of antimicrobial resistance and *Clostridioides difficile* infection. Other factors that support diminished use of very broad‐spectrum antibiotics include the fact that microbiological documentation is eventually available in virtually all episodes of pyelonephritis (therefore allowing therapy to be rapidly adjusted if initially inappropriate), and the fact that pyelonephritis is associated with a much lower risk of death than other invasive bacterial infections seen after solid organ transplantation.^9^, ^10^

In our survey, a 3rd‐generation cephalosporin monotherapy (using ceftriaxone or cefotaxime) was the preferred empirical regimen for the initial treatment of standard cases of post‐transplant pyelonephritis. This regimen, which was selected by almost 40% of survey participants globally, does not cover *P. aeruginosa* and *Enterococcus* spp. which are intrinsically resistant to these antibiotics. This practice does not follow current guidelines from the American Society of Transplantation Infectious Diseases Community of Practice, which suggest using anti‐pseudomonal ß‐lactams (i.e., cefepime, piperacillin‐tazobactam, or a carbapenem) in patients who require initial parenteral therapy, and restricting the use of narrower‐spectrum antibiotics such as ceftriaxone to patients with mild infection.^2^ Along the same line, Uptodate − which is a frequently used point‐of‐care clinical decision tool− currently states that kidney transplant recipients with pyelonephritis should receive empirical therapy covering both *P. aeruginosa* and *Enterococcus* spp. (e.g., using piperacillin‐tazobactam, meropenem, or cefepime‐vancomycin combination).^15^ Although systematically reviewing national and international guidelines on pyelonephritis was outside the scope of our study, not all of them recommend using anti‐pseudomonal antibiotics in kidney transplant recipients with pyelonephritis. Besides the question of systematically covering *P. aeruginosa* and *Enterococcus* spp. or not, it is critical to remember that resistance to third‐generation cephalosporins is also a feature of ESBL‐producing organisms which are a serious and growing threat in many transplant centers.^1^, ^2^, ^7^, ^10^

Whether *P. aeruginosa* should be systematically covered in all kidney transplant recipients with pyelonephritis is an important question that remains to be answered. While most respondents from some countries (Italy, India, and Bangladesh) reported systematically covering *P. aeruginosa*, this opinion was shared by less than 10% of respondents from some other countries (France, Belgium, the Netherlands, Switzerland, South Africa, and Singapore). Interestingly, available data indicate that *P. aeruginosa* explains less than 10% of all pyelonephritis episodes after kidney transplantation and that these *P. aeruginosa* infections typically occur in the presence of specific healthcare‐related risk factors.^1^, ^7^, ^12^, ^16^, ^17^ These data however come from Western Europe, and may not reflect the situation in other areas; in many countries, precise microbiological data are not available to guide initial antibiotic choice in kidney transplant recipients with pyelonephritis. We believe that the benefits and harms of systematically using very broad‐spectrum antibiotics that cover *P. aeruginosa* (and *Enterococcus* spp.) remain to be clarified among kidney transplant recipients with pyelonephritis, especially in patients who do not have sepsis or septic shock at the time of presentation. Importantly, the use of broad‐spectrum antibiotics is a key driver for antimicrobial resistance, which is a rapidly evolving and worrisome issue in transplantation, especially given the augmented risk of recurrent urinary tract infection.^7^, ^10^

Our survey indicates that recent urine culture results are used by most prescribers to guide initial antibiotic choice in kidney transplant recipients presenting with pyelonephritis. Specifically, in our survey, a recent culture result showing the growth of a resistant pathogen prompted clinicians to broaden the spectrum of antibiotic therapy (85% for ESBL‐producing organisms, 90% for carbapenemase‐producing organisms, and 94% for *P. aeruginosa*). The predictive value and usefulness of prior urine cultures remain to be determined, especially in patients who do not present with sepsis or septic shock and therefore appear to be at low risk of death.^10^, ^18^ Systematically covering recently identified microorganisms may increase the risk of antibiotic overuse. Interestingly, studies have shown that organisms causing post‐transplant pyelonephritis significantly differ genetically from those simply colonizing the urine of kidney transplant recipients.^19^ More practically, recent trials indicated that the historical practice of systematically screening for asymptomatic bacteriuria is not associated with improved outcomes in kidney transplant recipients^20^, ^21^, ^22^, ^23^, ^24^, ^25^; routine surveillance urine cultures are therefore being abandoned in many transplant centers, impacting prescribers’ ability to use their results to guide initial antibiotic choice when pyelonephritis occurs.

Overall, survey respondents attributed high importance to the appropriateness of initial empirical therapy in kidney transplant recipients with pyelonephritis. In particular, the vast majority of survey respondents considered the appropriateness of initial empirical antibiotic therapy as important to prevent death, and to improve other outcomes including long‐term kidney graft function. Respondents estimated an 8% 1‐month mortality risk for a kidney transplant recipient presenting with pyelonephritis, initially receiving inappropriate empirical therapy, and then receiving appropriate therapy after receipt of cultures results (interquartile range: 5%–20%). This estimated risk of death may seem high, especially given that our hypothetical vignette depicted a patient who did not have sepsis or septic shock at the time of presentation. Observational studies have reported much lower mortality rates in this situation (generally < 1%–2%), even in settings where inappropriateness of initial empirical therapy is common.^10^, ^26^ While rapid initiation of appropriate antibiotics is critical in patients who present with sepsis or septic shock, we believe that high‐quality studies are urgently needed to clarify the relationship between the appropriateness of initial empirical antibiotic therapy and patient and graft outcomes in non‐severe episodes of post‐transplant pyelonephritis.

Our study has notable strengths. First, we managed to capture practice and opinions from over 700 clinicians from 19 countries on six continents. Having national coordinators in all participating countries increased our ability to survey clinicians considered to be relevant to our research question. Capturing diverse perspectives and responses enhanced the generalizability and reliability of our survey results. Second, while other survey invitations are increasingly shared through social media, we instead decided to only distribute our invitation to a predetermined list of 2145 invitees. This allowed us to precisely determine the survey response rate (34%), which was higher than other international surveys focusing on transplant infectious diseases.^27^, ^28^, ^29^ Our study also has notable limitations. First, the non‐response rate of 66% may have significantly biased our findings. In the absence of detailed information on the characteristics of invited individuals who did not respond, this risk of bias is difficult to determine. Second, respondents were not questioned about the availability and use of local, national, and/or international guidelines. Third, the lack of precise microbiologic data from each participating region and hospital limited our ability to assess how local epidemiology correlated with practices. Last, the fact that our online questionnaire was only available in English might have dissuaded non‐English speaking clinicians from participating.

In conclusion, this global survey indicated that there is wide variability between and within countries in terms of initial empirical antibiotic therapy of post‐transplant pyelonephritis, that many patient‐level factors of unknown relevance are used by prescribers to guide initial antibiotic choice, and that many clinicians consider the appropriateness of initial empirical therapy to be critical to prevent death and other possible complications of pyelonephritis. High‐quality studies are needed to clarify the relationship between the appropriateness of initial empirical antibiotic therapy and important outcomes such as mortality and long‐term graft function in this population.

## AUTHOR CONTRIBUTIONS

**Julien Coussement**: Conceptualization; methodology; project administration; investigation; validation; formal analysis; visualisation; writing original draft. **Nassim Kamar** and **Camille N. Kotton**: Conceptualization; investigation; supervision; writing review–;editing. All other authors: Conceptualization; investigation; writing review–editing.

## CONFLICT OF INTEREST STATEMENT

The authors declare no conflict of interest.

## FUNDING INFORMATION

There was no specific funding for this survey.

## Supporting information

Supporting Information

## Data Availability

The data that support the findings of this study are available from the corresponding author upon reasonable request.
