# Supplementary material for: Initial empirical antibiotic therapy in kidney transplant recipients with pyelonephritis: A global survey of current practice and opinions across 19 countries on six continents
Source: Transpl Infect Dis. 2024 Aug 26;26(6):e14362. doi: 10.1111/tid.14362 (PMC11666887; doi:10.1111/tid.14362)
Supplement: Supplementary file 1 — Supporting Information [file TID-26-e14362-s001.pdf]

Supplementary appendix

Supplement to:

Coussement J, Bansal SB, Scemla A *et al.* Initial empirical antibiotic therapy in kidney transplant recipients with pyelonephritis: A global survey of current practice and opinions across nineteen countries on six continents.

This supplementary appendix has been provided by the authors to give readers additional information about their work.

Table S1. Number of survey respondents per country and survey response rate (countries ranked by number of survey respondents)

Country	Number of respondents	Number of invitations	Survey response rate
**India**	140	416	34%
**France**	90	178	51%
**United States**	83	349	24%
**Denmark**	45	137	33%
**Argentina**	42	83	51%
**Australia and New Zealand ***	42	340	12%
**Brazil**	36	84	43%
**Spain**	35	101	35%
**Israel**	33	105	31%
**Italy**	32	51	63%
**Belgium**	30	75	40%
**Netherlands**	27	34	79%
**Switzerland**	23	36	64%
**South Africa**	20	45	44%
**Singapore**	12	47	26%
**Bangladesh**	11	38	29%
**Czech Republic**	11	15	73%
**Austria**	9	11	82%
**Total (globally)**	**721**	**2145**	**34%**

* Including 36 respondents from Australia and 6 respondents from New Zealand (invitations sent collectively in these two countries)

Table S2. Characteristics of the 721 survey respondents

**Specialty**	
Nephrology	68% (n=491)
Infectious diseases (including Transplant infectious diseases)	29% (n=208)
Other *	3% (n=22)
**Level of medical experience**	
Junior doctor in specialist training	2% (n=12)
Specialist for < 5 years	15% (n=109)
Specialist for 5-20 years	51% (n=365)
Specialist for > 20 years	33% (n=235)
**Type of hospital**	
Academic / university hospital	76% (n=551)
Non-academic private hospital	14% (n=98)
Non-academic public hospital	10% (n=72)
**Number of kidney transplants performed last year in the hospital**	
Less than 50	28% (n=203)
50 to 100	29% (n=212)
More than 100	30% (n=214)
Center where transplant recipients are followed-up, but no transplants performed	13% (n=92)

***** Including transplant surgeons (n=20) and intensive care unit physicians (n=2)

Table S3. Variation in practice and opinions between and within countries, in terms of initial empirical antibiotic management of pyelonephritis in kidney transplant recipients who do not require intensive care unit admission (countries ranked by number of survey respondents)

Country (with number of participants)	Preferred initial empirical regimen		Preferred initial empirical monotherapies			Opinion that following microorganisms should be systematically covered	
	**One antibiotic**	**Two antibiotics**	**First preferred**	**Second preferred**	**Third preferred**	***Pseudomonas aeruginosa***	***Enterococcus* spp**
**India** (n=140)	74% (103/140)	26% (37/140)	Cefoperazone/ Sulbactam (38/103)	Piperacillin/ tazobactam (25/103)	Carbapenem (18/103)	51% (66/130)	17% (21/125)
**France** (n=90)	96% (86/90)	4% (4/90)	3GC (ceftriaxone or cefotaxime) (80/86)	Piperacillin/ Tazobactam (4/86)	Other (2/86)	3% (3/86)	1% (1/85)
**USA** (n=83)	88% (73/83)	12% (10/83)	3GC (ceftriaxone or cefotaxime) (32/73)	Cefepime (24/73)	Piperacillin/ tazobactam (13/73)	31% (25/80)	14% (11/78)
**Denmark** (n=45)	89% (40/45)	11% (5/45)	Piperacillin/ tazobactam (36/45)	Other (9/45)	N/A	33% (14/42)	26% (11/42)
**Argentina** (n=42)	95% (40/42)	5% (2/42)	Piperacillin/ tazobactam (10/40)	Carbapenem (9/40)	3GC (ceftriaxone or cefotaxime) (7/40)	21% (8/38)	11% (4/38)
**Australia and NZ *** (n=42)	81% (34/42)	19% (8/42)	3GC (ceftriaxone or cefotaxime) (15/34)	Piperacillin/ tazobactam (13/34)	2nd generation cephalosporin (3/34)	17% (7/42)	19% (8/42)
**Brazil** (n=36)	100% (36/36)	0% (0/36)	3GC (ceftriaxone or cefotaxime) (23/36)	Fluoroquinolone (3/36)	Other (10/36)	23% (8/35)	14% (5/35)
**Spain** (n=35)	91% (32/35)	9% (3/35)	3GC (ceftriaxone or cefotaxime) (19/32)	Piperacillin/ tazobactam (5/32)	Carbapenem (3/32)	18% (6/34)	9% (3/34)
**Israel** (n=33)	91% (30/33)	9% (3/33)	3GC (ceftriaxone or cefotaxime) (13/30)	Piperacillin/ tazobactam (11/30)	Aminoglycoside monotherapy (4/30)	24% (7/29)	10% (3/29)
**Italy** (n=32)	91% (29/32)	9% (3/32)	Piperacillin/ tazobactam (20/29)	3GC (ceftriaxone or cefotaxime) (3/29)	Fluoroquinolone (3/29)	53% (16/30)	23% (7/30)
**Belgium** (n=30)	100% (30/30)	0% (0/30)	Temocillin (8/30)	3GC (ceftriaxone or cefotaxime) (8/30)	Amoxicillin/ clavulanic acid (6/30)	4% (1/28)	11% (3/28)
**Netherlands** (n=27)	100% (27/27)	0% (0/27)	3GC (ceftriaxone or cefotaxime) (18/27)	2nd generation cephalosporin (8/27)	Fluoroquinolone (1/27)	4% (1/26)	12% (3/26)
**Switzerland** (n=23)	100% (23/23)	0% (0/23)	3GC (ceftriaxone or cefotaxime) (17/23)	Piperacillin/ tazobactam (4/23)	Other (2/23)	4% (1/23)	18% (4/22)
**South Africa** (n=20)	90% (18/20)	10% (2/20)	3GC (ceftriaxone or cefotaxime) (5/18)	Amoxicillin/ clavulanic acid (5/18)	Fluoroquinolone (3/18)	6% (1/17)	24% (4/17)
**Singapore** (n=12)	100% (12/12)	0% (0/12)	3GC (ceftriaxone or cefotaxime) (11/12)	Amoxicillin/ clavulanic acid (1/12)	N/A	8% (1/12)	8% (1/12)
**Bangladesh** (n=11)	82% (9/11)	18% (2/11)	Carbapenem (6/9)	Other (3/9)	N/A	60% (6/10)	50% (5/10)
**Czech Republic** (n=11)	82% (9/11)	18% (2/11)	3GC (ceftriaxone or cefotaxime) (5/9)	Carbapenem (2/9)	Other (2/9)	20% (2/10)	20% (2/10)
**Austria** (n=9)	100% (9/9)	0% (0/9)	Amoxicillin/ clavulanic acid (5/9)	Piperacillin/ tazobactam (2/9)	Other (2/9)	22% (2/9)	11% (1/9)

3GC: 3rd generation cephalosporin. N/A: not applicable. NZ: New Zealand. USA: United States of America.

* Invitations sent collectively in these two countries.

Table S4. Variation in practice and opinions between nephrologists and infectious diseases physicians, in terms of initial empirical antibiotic management of pyelonephritis in kidney transplant recipients who do not require intensive care unit admission

	Nephrologists (n = 491)	Infectious diseases physicians (n = 208)
**Preferred initial empirical regimen**		
One antibiotic	88% (432/491)	91% (189/208)
Two antibiotics	12% (59/491)	9% (19/208)
**Preferred initial empirical single-drug regimen**		
3rd generation cephalosporin	40% (171/432)	48% (91/189)
Piperacillin/tazobactam	22% (94/432)	24% (45/189)
**Opinion that *Pseudomonas aeruginosa* should be covered …**		
Systematically (in all patients with post-transplant pyelonephritis)	27% (126/459)	22% (44/204)
If recent urine culture* showed significant growth of *P. aeruginosa*	94% (431/459)	95% (194/204)
**Opinion that *Enterococcus* spp. should be covered …**		
Systematically (in all patients with post-transplant pyelonephritis)	16% (70/450)	9% (19/204)
If recent urine culture* showed significant growth of *Enterococcus* spp.	86% (388/450)	82% (168/204)
**Opinion that initiating antibiotic(s) that are active while awaiting the results of cultures is important to…**		
Reduce time to resolution of fever	93% (405/435)	90% (181/202)
Reduce risk of progression to septic shock or renal abscess	98% (426/435)	93% (188/202)
Reduce duration of hospital stay	93% (403/435)	88% (178/202)
Reduce mortality	88% (383/435)	83% (168/202)
Improve long-term kidney graft function	80% (350/435)	75% (152/202)
**Estimated risk of 30-day mortality in patients initially receiving inappropriate empirical therapy but then receiving appropriate antibiotic therapy once urine and blood cultures results are provided**		
Median [interquartile range]	5% [3-20]	10% [5-15]

* Defined as a urine culture from last 3-6 months

Paper version of the survey questionnaire.

Preliminary comments:

This survey should take approximately 5 minutes to complete. It is about the empirical antibiotic management of pyelonephritis in kidney transplant recipients (i.e., prior to the receipt of culture and susceptibility testing results). If you do not personally take care of adult kidney transplant recipients with pyelonephritis on a regular basis, please do not participate in this survey.

Demographic parameters:

What country do you practice in?

☐ Argentina
☐ Australia
☐ Austria
☐ Bangladesh
☐ Belgium
☐ Brazil
☐ Canada
☐ Czech Republic
☐ Denmark
☐ France
☐ Germany
☐ India
☐ Israel
☐ Italy
☐ Netherlands
☐ New Zealand
☐ Singapore
☐ South Africa
☐ Spain
☐ Switzerland
☐ United States
☐ Other: specify____

What is your specialty?

- Nephrology (or Clinical Transplantation)
- Infectious Diseases (including Transplant Infectious Diseases)
- Transplant Surgery
- Other: specify: _______________

What is your level of medical experience?

- Junior doctor in specialty training (i.e., I have graduated from medical school)
- Specialist (for example, nephrologist) for < 5 years
- Specialist (for example, nephrologist) for 5-20 years
- Specialist (for example, nephrologist) for > 20 years

How would you describe the hospital where you mainly practice?

- Academic / university hospital
- Non-academic public hospital
- Non-academic private hospital
- Other: specify: _______________

Approximately, how many kidney transplants were performed last year in this hospital?

- 0 (I work in a peripheral centre where transplant recipients are followed-up, but no transplants are performed)
- Less than 50
- 50-100
- More than 100

To answer the following questions, consider the hypothetical case of a kidney transplant recipient who is admitted to your hospital with fever, dysuria and graft pain, and is diagnosed with probable graft pyelonephritis. Two sets of blood cultures and a urine sample are collected, and you then decide to initiate empiric antibiotic therapy while awaiting the results of cultures. The patient does not require intensive care levels of support, and there are no recent culture results to guide antibiotic choice.

1. **What is your preferred empirical antibiotic therapy whilst awaiting culture results?**

- **Monotherapy (i.e., one antibiotic only):**
    - o Amoxicillin
    - o Amoxicillin/clavulanic acid
    - o Fluoroquinolone (e.g., ciprofloxacin, ofloxacin, or levofloxacin)
    - o Trimethoprim/sulfamethoxazole
    - o 1st generation cephalosporin (e.g., cefazolin)
    - o 2nd generation cephalosporin (e.g., cefuroxime)
    - o 3rd generation cephalosporin (e.g., ceftriaxone or cefotaxime)
    - o 4th generation cephalosporin (i.e., cefepime)
    - o Aztreonam
    - o Temocillin
    - o Ticarcillin
    - o Ticarcillin/clavulanic acid
    - o Piperacillin
    - o Piperacillin/tazobactam
    - o Meropenem or imipenem
    - o Ertapenem
    - o Ceftazidime/avibactam
    - o Ceftolozane/tazobactam
    - o Aminoglycoside monotherapy (e.g., gentamicin or amikacin)
    - o Other antibiotic: ___________

- **Dual therapy (i.e., two antibiotics):**

First agent:

- o Amoxicillin
- o Amoxicillin/clavulanic acid
- o Fluoroquinolone (e.g., ciprofloxacin, ofloxacin, or levofloxacin)
- o Trimethoprim/sulfamethoxazole
- o 1st generation cephalosporin (e.g., cefazolin)
- o 2nd generation cephalosporin (e.g., cefuroxime)
- o 3rd generation cephalosporin (e.g., ceftriaxone or cefotaxime)
- o 4th generation cephalosporin (i.e., cefepime)
- o Aztreonam
- o Temocillin
- o Ticarcillin
- o Ticarcillin/clavulanic acid
- o Piperacillin
- o Piperacillin/tazobactam
- o Meropenem or imipenem
- o Ertapenem
- o Ceftazidime/avibactam
- o Ceftolozane/tazobactam
- o Other first agent: ___________

Second agent:

- o Aminoglycoside (e.g., gentamicin or amikacin)
- o Glycopeptide (e.g., vancomycin or teicoplanin)
- o Daptomycin
- o Linezolid
- o Fluoroquinolone (e.g., ciprofloxacin, ofloxacin, or levofloxacin)
- o Other second agent: ____________

2. **Would any of the following scenarios make you select a regimen that has a broader spectrum than the one you selected in the previous question?** Please answer "YES" or "NO" to each of the following.
   - Septic shock or sepsis (typically requiring intensive care unit admission)
   - Imaging done at admission showing urinary tract obstruction
   - Imaging done at admission showing abscess of the kidney graft
   - Recent transplant (in last 3-6 months)
   - Healthcare-associated infection (defined as urinary catheter, hospital stay and/or haemodialysis in last 3-6 months)
   - Recent antibiotic treatment (in last 3-6 months)
   - Current use of trimethoprim/sulfamethoxazole prophylaxis
   - Current use of another antibiotic to prevent urinary tract infections (e.g., nitrofurantoin, fosfomycin, or cephalexin)
   - Recent urine culture with growth of an ESBL-producing organism (in last 3-6 months)
   - Recent urine culture with growth of a carbapenemase-producing organism (in last 3-6 months)

3. **Should empirical therapy cover *Pseudomonas aeruginosa* (for instance using piperacillin-tazobactam, cefepime or carbapenem) in kidney transplant recipients who present with symptoms of pyelonephritis (e.g., fever, dysuria, and graft pain) but do not require intensive care unit admission?** Multiple responses allowed.
   - Yes (systematically for all hospitalised patients)
   - No (never)
   - In the following situation: recent urine culture with significant growth of *Pseudomonas aeruginosa* (in last 3-6 months)
   - In the following situation: imaging done at admission showing focal complication (urinary tract obstruction, or abscess of the kidney graft)
   - In the following situation: kidney transplantation in last 3-6 months
   - In the following situation: healthcare-associated infection (defined as urinary catheter, hospital stay or haemodialysis in last 3-6 months)
   - In the following situation: receipt of antibiotic therapy in last 3-6 months
   - In the following situation: current receipt of trimethoprim/sulfamethoxazole prophylaxis
   - In the following situation: current receipt of another antibiotic to prevent urinary tract infections (e.g., nitrofurantoin, fosfomycin, or cephalexin)

4. **Should empirical therapy cover *Enterococcus* spp. in kidney transplant recipients who present with symptoms of pyelonephritis (e.g., fever, dysuria, and graft pain) but do not require intensive care unit admission?** Multiple responses allowed.
   - Yes (systematically for all hospitalised patients)
   - No (never)
   - In the following situation: recent urine culture with significant growth of *Enterococcus* spp. (in last 3-6 months)
   - In the following situation: imaging done at admission showing focal complication (urinary tract obstruction, or abscess of the kidney graft)
   - In the following situation: kidney transplantation in last 3-6 months
   - In the following situation: healthcare-associated infection (defined as urinary catheter, hospital stay or haemodialysis in last 3-6 months)
   - In the following situation: receipt of antibiotic therapy in last 3-6 months
   - In the following situation: current receipt of trimethoprim/sulfamethoxazole prophylaxis
   - In the following situation: current receipt of another antibiotic to prevent urinary tract infections (e.g., nitrofurantoin, fosfomycin, or cephalexin)

Let's go back to the case of the kidney transplant recipient admitted with fever and other symptoms of pyelonephritis. The patient does not require intensive care levels of support. Blood and urine samples are collected (results pending).

5. **What is your estimated risk of 30-day mortality if this patient initially receives inappropriate empiric antibiotic therapy (i.e., antibiotic[s] which are not active against the causative pathogen), but then receives appropriate therapy once urine and blood culture results are provided?** You can give any number between 0 and 100%.

6. **Do you believe that ensuring that this patient is started on initial antibiotic(s) that are active while awaiting the results of cultures is important to…**
   - … reduce time to resolution of fever?
   - … reduce the risk of progression to complications such as septic shock or renal abscess?
   - … reduce duration of hospital stay?
   - … reduce mortality?

   (*for each answer:** ***I strongly believe so / I somewhat believe so/ I don't believe so / I don't know)
